# Supplementary material for: Phosphoproteomic-based kinase profiling early in influenza virus infection identifies GRK2 as antiviral drug target
Source: Nat Commun. 2018 Sep 11;9:3679. doi: 10.1038/s41467-018-06119-y (PMC6133941; doi:10.1038/s41467-018-06119-y)
Supplement: Supplementary file 1 — Supplementary Information [file 41467_2018_6119_MOESM1_ESM.pdf]

Phosphoproteomic-based kinase profiling early in influenza virus infection identifies GRK2 as antiviral drug target

Yángüez et al.

Supplementary information

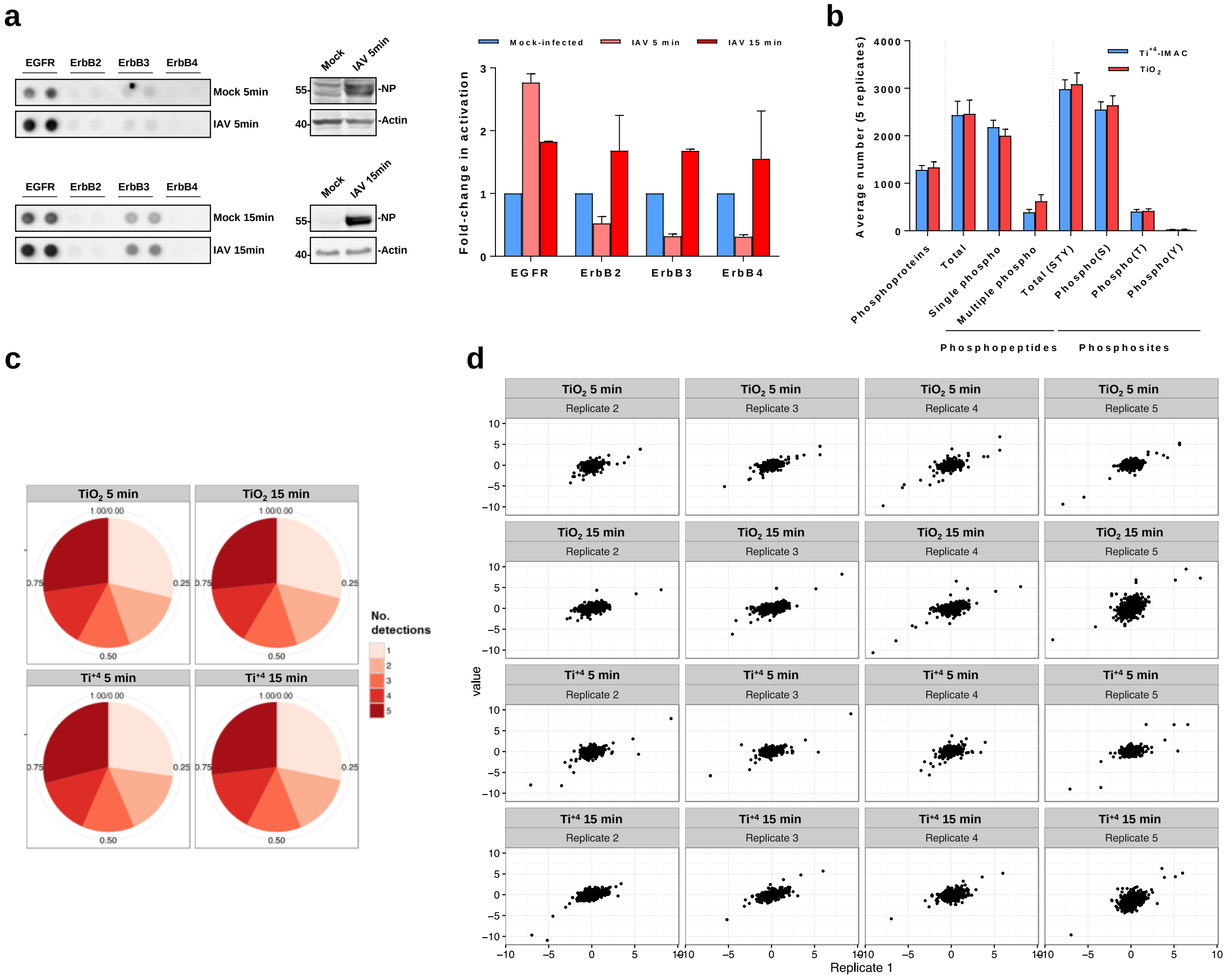

Supplementary Figure 1. Statistics and quality control for phosphopeptide enrichment and detection. (a) EGFR phosphorylation in response to IAV infection. Serum-starved A549 cells were infected for 5 or 15 min with IAV A/WSN/33 (H1N1) at high multiplicity of infection (MOI=25 PFU/cell) or left uninfected and the activation of different receptor tyrosine kinases (RTKs) was assessed using a Proteome Profiler Human Phospho-RTK Array Kit (R&D Systems). Raw pictures for the spots corresponding to EGFR and other members of the ErbB family are shown together with a quantification of the signal intensities from three replicates. Error bars represent standard deviation. **(b)** Number of identifications and comparison of the phospho-enrichment efficiency of TiO_2 or Ti^{+4} chromatographies (Scaffold PTM 3.0) from five replicates. Error bars represent standard deviation**. (c)** Number of biological replicates in which a particular phosphopeptide is detected. In all cases, more than 70% of all phosphopeptides were detected at least twice, with at least 25% being detected in all five replicates. **(d)** Log correlation plots of peptide intensities across replicates indicate high-interexperiment replicability. All replicates were included in the analyses with the exception of replicate 5 at 15 min for both IMAC and TiO_2 setups.

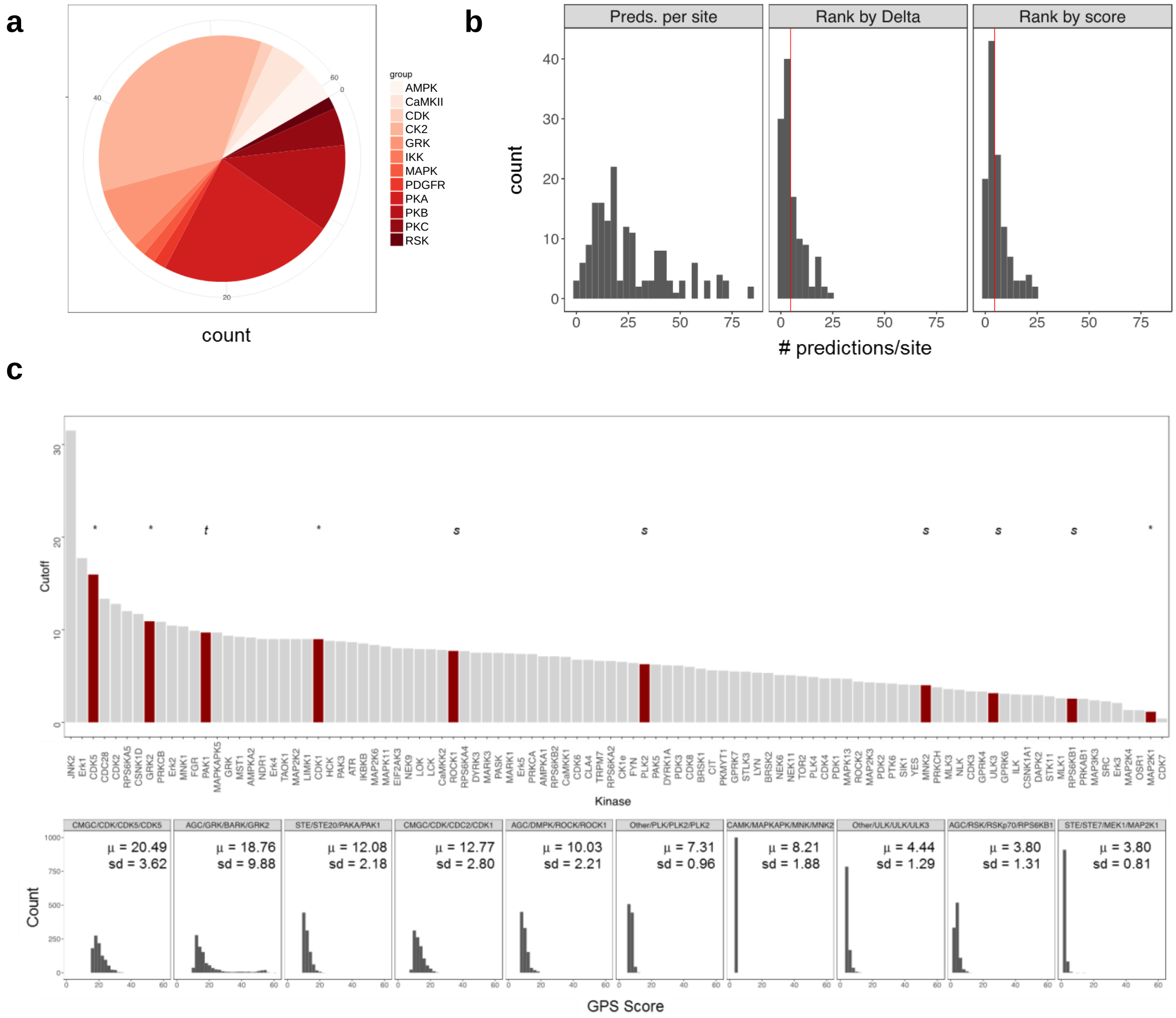

Supplementary Figure 2. Optimization of kinase prediction. (a) To optimize the settings for selecting results from high-confidence GPS 3.0 predictions, we performed predictions on selected kinase-phosphosite pairs implicated in viral infection from the gold standard, Phospho.ELM database (no. proteins = 51, no. of unique sites = 160). **(b)** We subsequently checked which filter settings we needed to use in order to extract the gold standard kinase-phosphosite pairs from all other predictions in terms of the parameters provided by GPS 3.0, score and cut-off, and a derived parameter (Delta), which is the difference between the score and the cut-off. A median of 19 kinases or kinase families can be predicted for each of the 160 sites; of these, 118 (74%) included a prediction for the correct kinase. Approximately 70% of the correct predictions are in the top five hits in terms of either the program-provided score or the derived Delta parameter. **(c)** GPS 3.0 kinases ranked by cut-off. Top predicted kinases for phosphopeptides in early IAV infection are shown in red; significantly enriched peptides (p.val < 0.05) are indicated with asterisks. Distributions of GPS 3.0 scores for each of these kinases are shown in the lower panel.

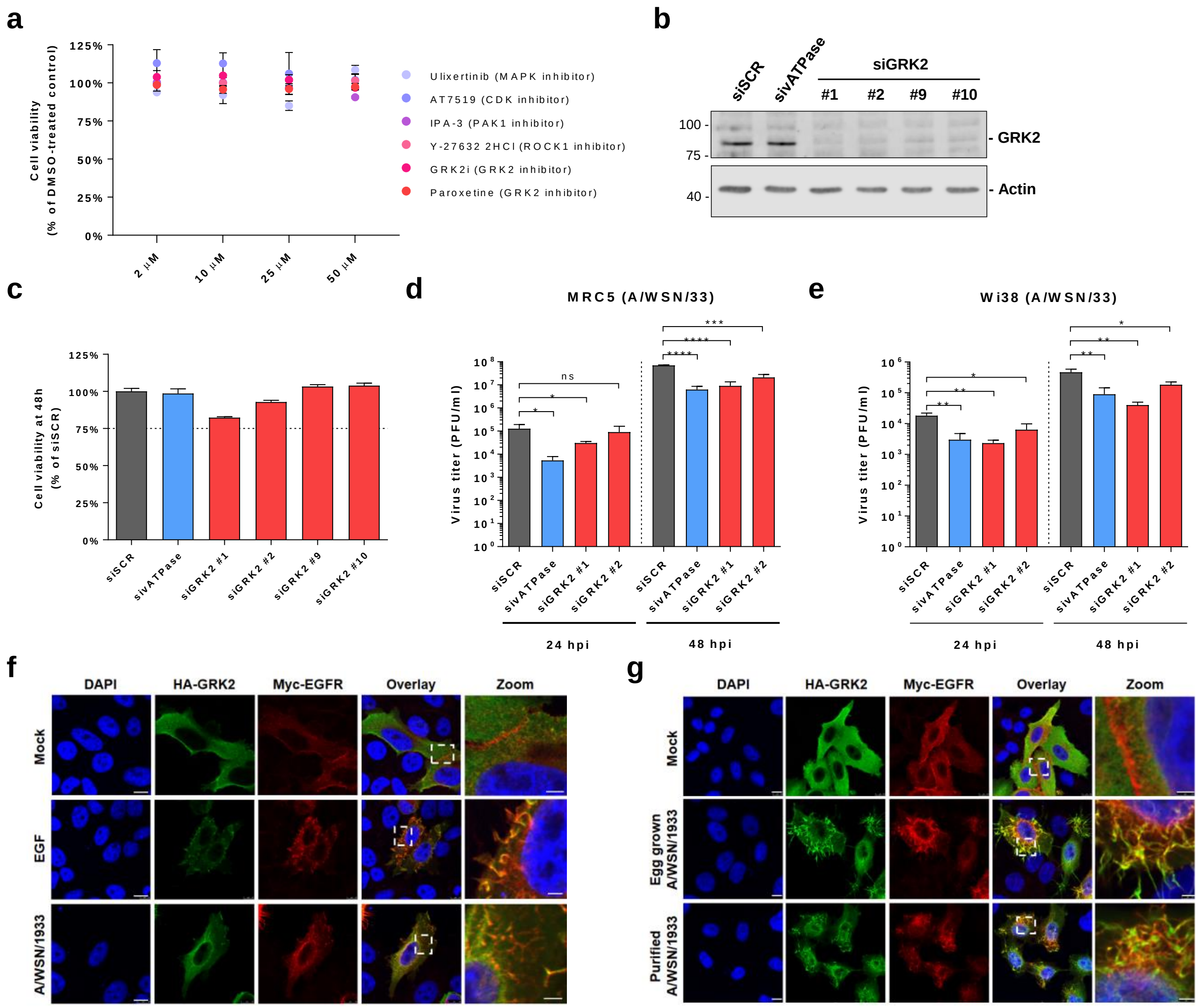

Supplementary Figure 3. GRK2 is a proviral host factor that becomes activated within minutes of IAV infection. (a) Cytotoxicity of the different kinase inhibitors. A549 cells were incubated in presence of the indicated amounts of the different kinase inhibitors for 13 h. Cell viability was determined using the CellTiter-Glo assay (Promega) and is shown as a percentage of DMSO-treated control cells. Mean values from triplicates are shown. **(b-c)** Silencing efficiency and cytotoxicity. A549 cells were transfected with a control siRNA (siSCR), an siRNA targeting vATPase required for viral entry, or different siRNAs targeting GRK2. At 48 h post-transfection, the amount of GRK2 was evaluated by western blot (b) or cell viability in the different conditions was evaluated in parallel using the CellTiter-Glo assay (Promega) and is shown as a percentage of siSCR control (c). Mean values from triplicates are depicted. **(d-e)** IAV replication in GRK2-silenced MRC5 (d) or Wi38 (e) cells. Cells were transfected with a control siRNA (siSCR), a siRNA targeting vATPase, or different siRNAs targeting GRK2. At 48 h post-transfection, cells were infected with A/WSN/33 (H1N1, MOI=0.01 PFU/cell), supernatants were collected at the indicated time post-infection and virus titers from three replicates were determined by plaque assay. Statistical significance was determined by unpaired t test: ns (non-

significant) = P > 0.05; * = P ≤ 0.05; ** = P ≤ 0.01; *** = P ≤ 0.001; **** P ≤ 0.0001. **(f)** Hep-2 cells were transfected with HA-GRK2 and myc-EGFR before being infected with A/WSN/1933 (H1N1, MOI=100 PFU/cell) or stimulated with recombinant human epidermal growth factor (EGF) for 5 min at 37°C. Cells were fixed and stained using antibodies against the HA-tag (green), EGFR (red) and DAPI (blue). Cellular localization of the proteins was analyzed using confocal microscopy. Scale bars correspond to 10 µm, for zoom images scale bars correspond to 2.5 µm. **(g)** Experimental set-up as in f but the infection was performed with either unpurified ("egg grown A/WSN/33") or purified virus stocks ("Purified A/WSN/33") for 15 min. Scale bars correspond to 10 µm, for zoom images scale bars correspond to 2.5 µm. For all panels, error bars represent standard deviation.

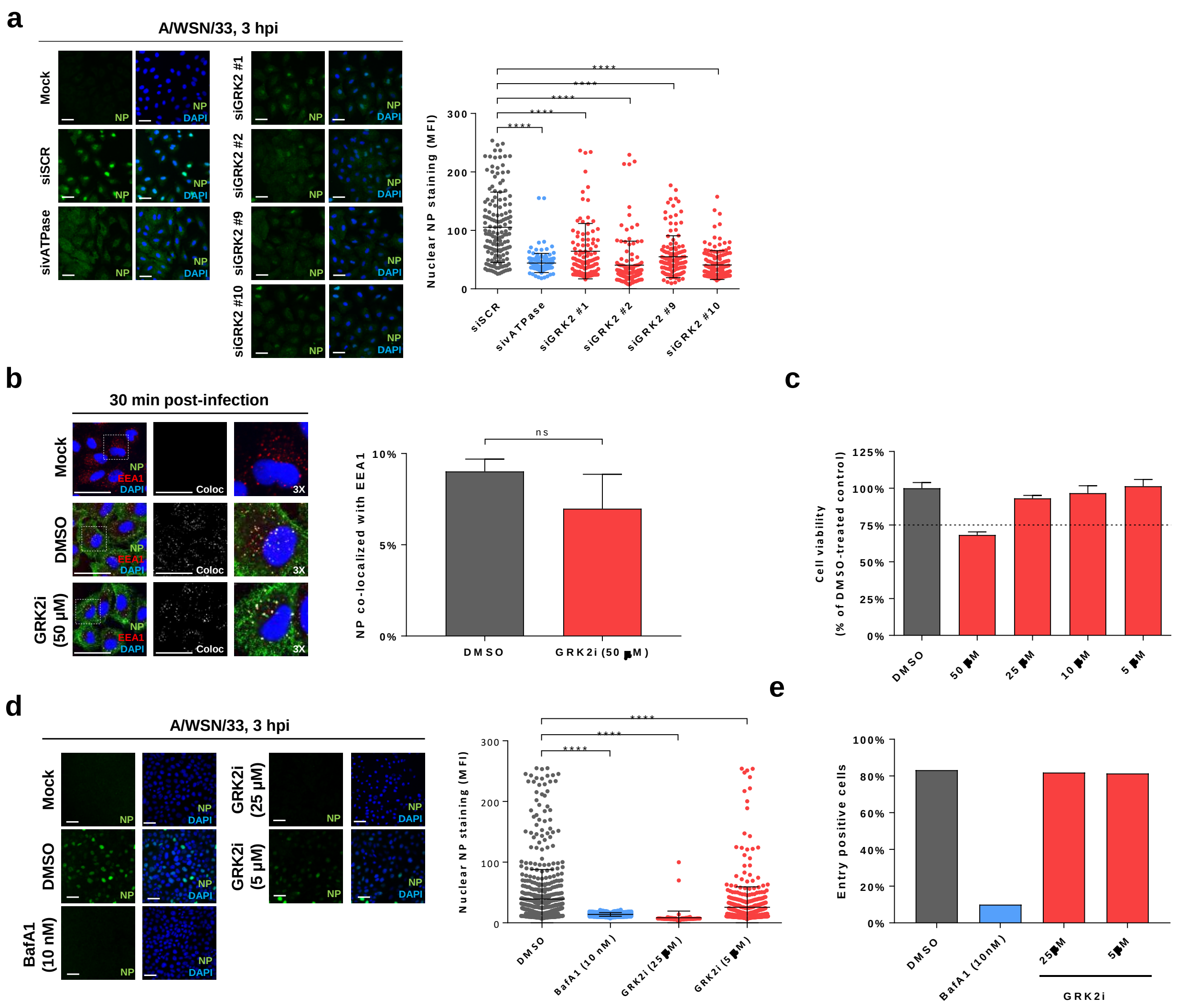

Supplementary Figure 4. Quantitative analysis of the different steps of IAV entry. (a) Effect of GRK2 silencing on the expression and localization of NP. Cells were transfected with siRNAs. At 48 h post-transfection, cells were infected on ice with IAV A/WSN/33 (H1N1, MOI=5 PFU/cell) for 1 h to synchronize the infection. Cells were incubated at 37°C for additional 3 h, fixed and stained for viral nucleoprotein (NP, green) and nuclei (DAPI, blue). Representative images are shown (scale bar corresponds to 40 µm). The mean fluorescence green intensity (MFI) in each cell (n > 200) was quantified using ImageJ. One representative experiment out of four independent experiments is shown. **(b)** A549 cells were treated with the indicated compounds for 1 h and infected with A/WSN/34 (H1N1) with an MOI=25 PFU/cell. At 30 min post-infection, cells were fixed and stained for viral nucleoprotein (NP, green), early endosomes (EEA1, red) and the nuclei were stained with DAPI (blue) and images were acquired by confocal microscopy (scale bar corresponds to 10 µm). The co-localization of NP (viral particles) with EEA1 (early endosomes), shown in the pictures in black and white, was determined using Imaris and it is shown as percentage of the total NP signal. One representative experiment out of three independent experiments is shown. **(c)** Cytotoxicity of GRK2i in MDCK cells. MDCK cells were incubated in the presence of the indicated amounts of the

kinase inhibitor for 5 h. Cell viability was determined using the CellTiter-Glo assay (Promega) and is shown as a percentage of DMSO-treated control cells. Mean values from triplicates are depicted. **(d)** MDCK cells were pre-treated with the indicated compounds and infected on ice with IAV A/WSN/33 (H1N1, MOI=5 PFU/cell) for 1 h to synchronize the infection. Cells were incubated at 37°C for additional 3 h in the presence of the compounds, fixed and stained for viral nucleoprotein (NP, green) and the nuclei were stained with DAPI (blue). Representative images are shown (scale bar corresponds to 40 µm). The mean fluorescence green intensity (MFI) in each cell (n > 200) was quantified using ImageJ. One representative experiment out of three independent experiments is shown. **(e)** MDCK cells were pre-treated for 1 h with the indicated concentrations of the GRK2 inhibitor and infected with IAV virus-like particles (VLPs) that contain HA and NA from A/WSN/33 and harbour a beta-lactamase reporter protein fused to the influenza matrix protein-1 (BlaM1). At 4 h pi in the presence of the inhibitor, the emission of the fluorogenic substrate CCF-2, which is a direct measurement of viral fusion, was determined by flow cytometry. For all panels, error bars represent standard deviation and statistical significance was determined by unpaired t test: ns (non-significant) = $P > 0.05$; * = $P \leq 0.05$; ** = $P \leq 0.01$; *** = $P \leq 0.001$; **** $P \leq 0.0001$.

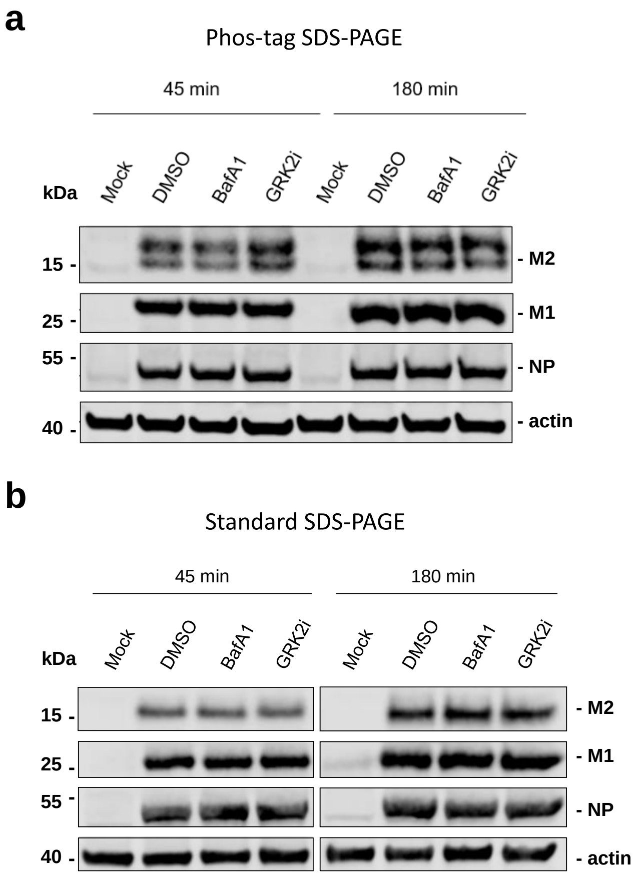

Supplementary Figure 5. Phosphorylation status of viral proteins upon GRK2 inhibition. (a) A549 cells were serum-starved for 16 h, treated with cycloheximide and GRK2i (50 µM), bafilomycin A1 (10 nM) or DMSO (25 µM) for 1 h at 37°C and then infected on ice with A/WSN/1933 (H1N1, MOI=100 PFU/cell) in presence of the inhibitors. After 1 h the inoculum was removed and replaced by piDMEM supplemented with the inhibitors and cycloheximide using the indicated concentrations. Cells were lysed after an incubation time of 45 min or 3 h at 37°C. Samples were run on a Zn^{2+} phostag gel and analyzed by western blot. **(b)** Same experimental set up but samples were analyzed by standard SDS-PAGE and western blot.

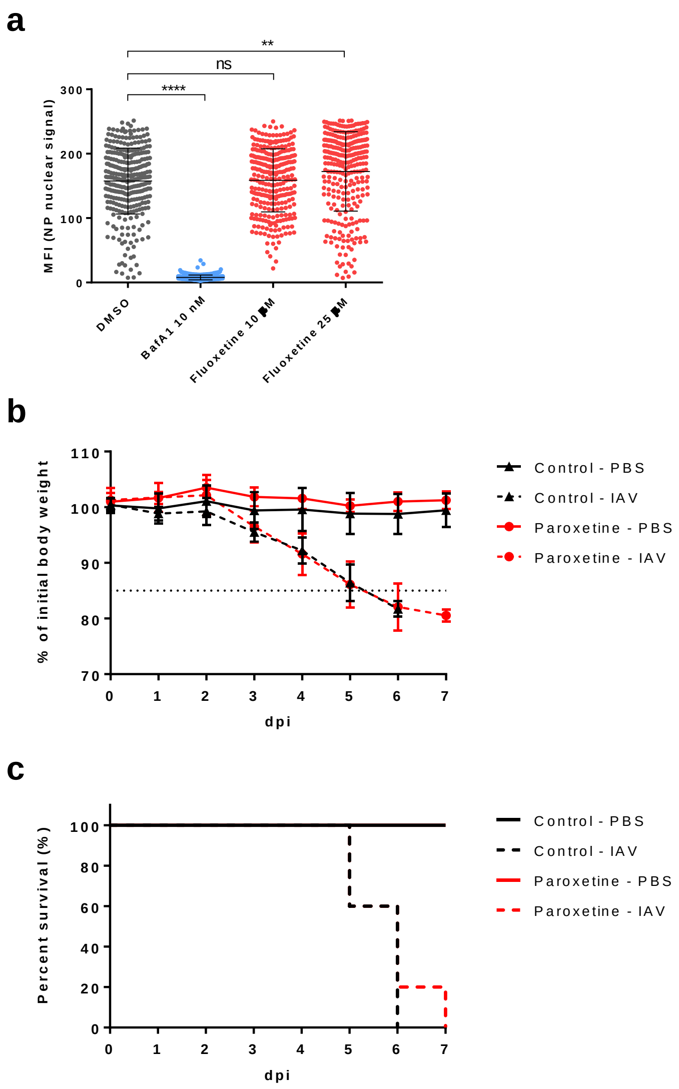

Supplementary Figure 6. Paroxetine limits IAV replication in human airway epithelium cultures and in mice. (a) A549 cells were treated with the indicated concentrations of DMSO, bafilomycin A1 or Fluoxetine for 1 h at 37°C. Then, cells were infected on ice with IAV A/WSN/33 (H1N1) at MOI=25 PFU/cell for 1 h followed by an incubation at 37°C for 4 h in the presence of the compounds. Cells were fixed and viral nucleoprotein (NP) and cell nuclei were stained. The distribution of the NP signal was analysed by confocal microscopy and the mean fluorescence intensity (MFI) in the cell nuclei (n > 200) was quantified using Image J. One representative experiment out of two independent experiments is shown. Unpaired t test was used for statistical analysis: **** = p-value < 0.0001; * = p-value < 0.01; ns = p-value > 0.05. **(b-c)** 9 week old C57BL/6J mice were injected i.p. with 5 mg/kg paroxetine in 5% DMSO in PBS or 5% DMSO in PBS (solvent) 24 h prior to infection. Mice were infected with 10 PFU of A/Netherlands/602/2009 (pdmH1N1) or inoculated with PBS via the intranasal route. Paroxetine or solvent treatment was repeated on d0, d1 and d2 pi. Mice were monitored daily for weight loss (b) and survival (c) up to d7 when all infected mice reached humane endpoints. For all panels, error bars represent standard deviation.

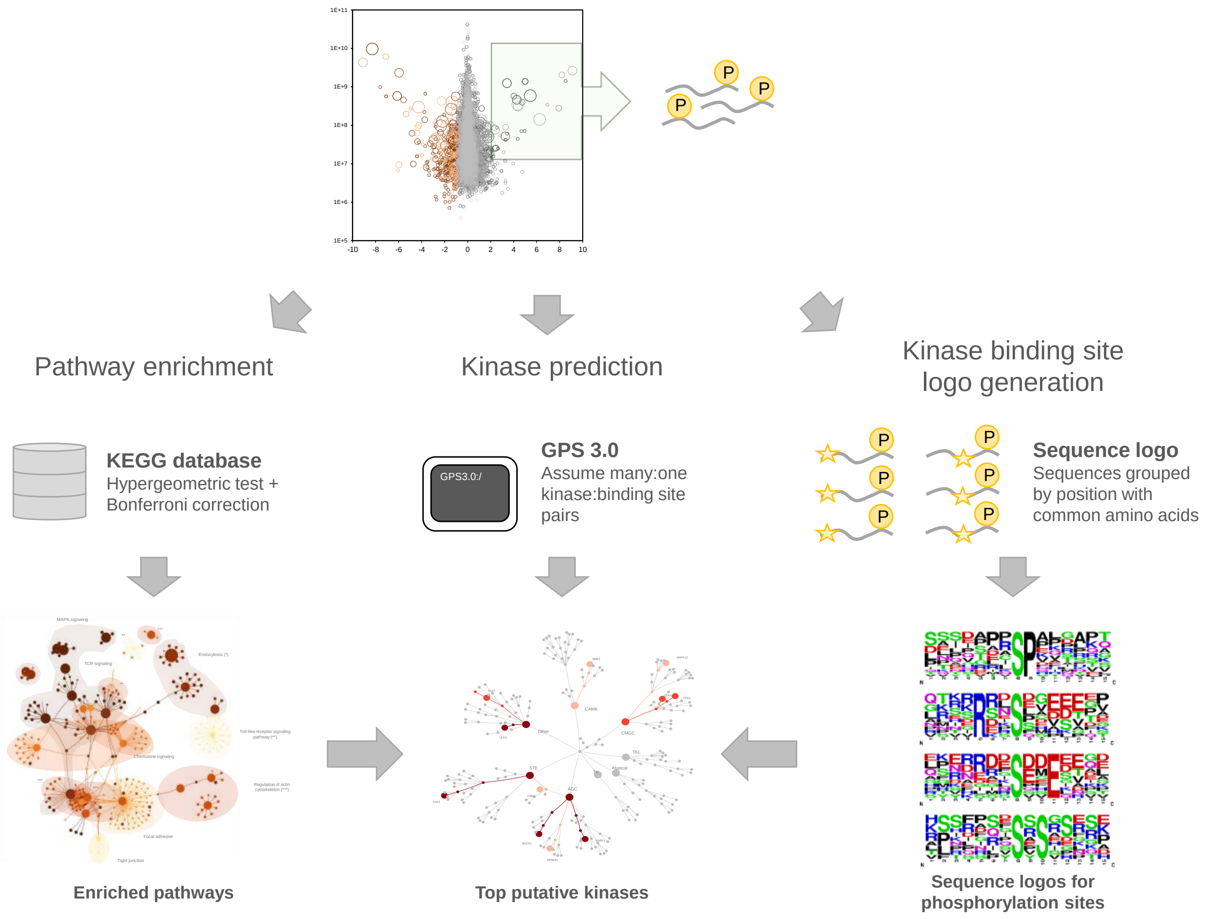

Supplementary Figure 7. General overview of the bioinformatics analysis.

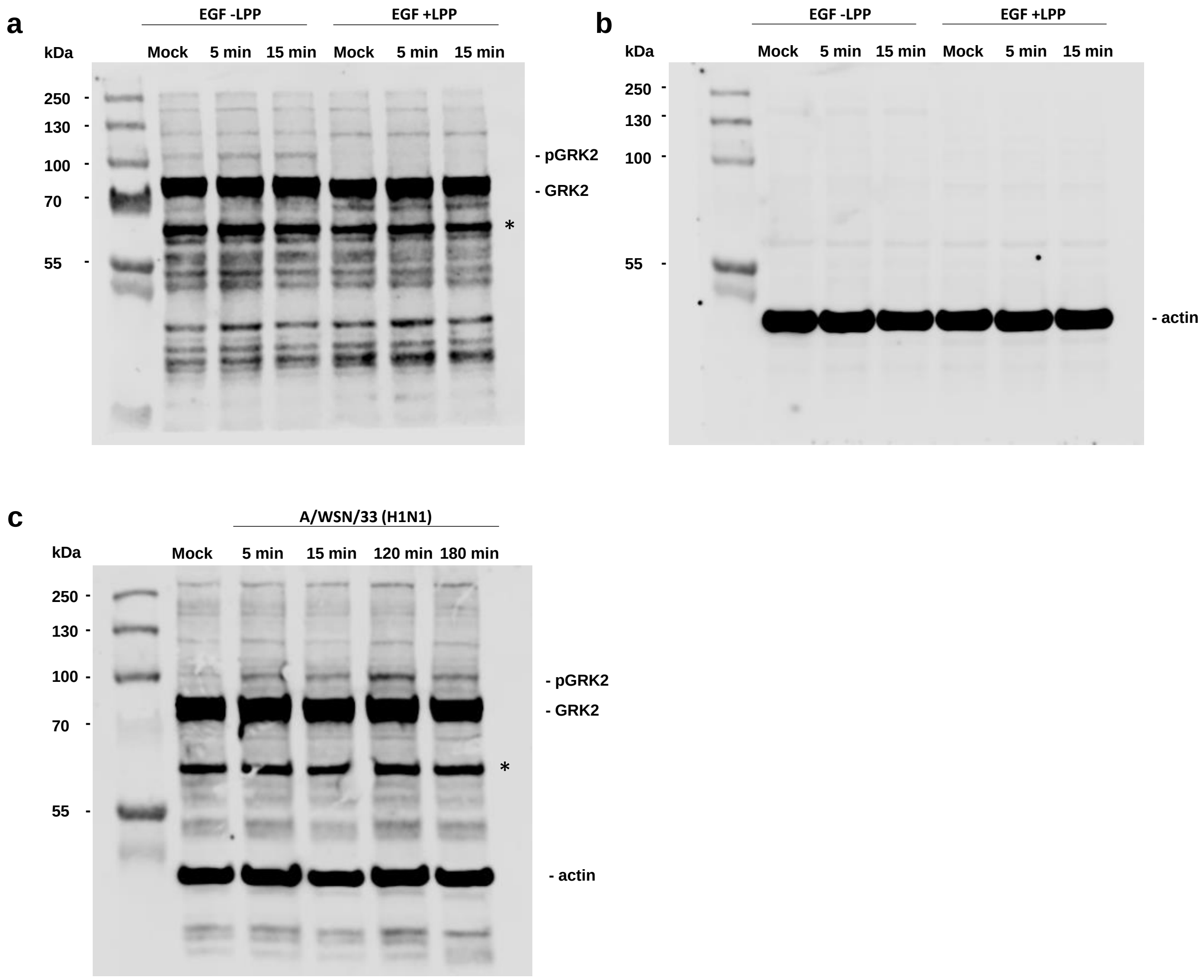

Supplementary Figure 8. Uncropped blots from Figures 3d-e. (a-b) Uncropped blots of Figure 3d. (a) Blot stained with an antibody against GRK2 and a fluorescent secondary antibody with an excitation length of 800 nm. The band indicated by an “*” is unspecific. (b) Staining for β-actin on the same blot using a fluorescent secondary antibody with an excitation length of 680 nm. **(c)** Uncropped blot of Figure 3e stained with antibodies against GRK2 and β-actin and a fluorescent secondary antibody from Li-Cor with an excitation length of 680nm. The band indicated by an “*” is unspecific.

Perc.match	Adj.p.val	KEGG names	Signature size	Matched genes in signature
0.037735849	2.29E-08	MAPK signaling pathway	265	RAF1,ATF2,RPS6KA4,AKT3,HSPB1,MAP2K2,PLA2G4A,MAP3K7,FLNB,CRK
0.035532995	1.63E-06	Endocytosis	197	MDM2,RAB11FIP5,ZFYVE16,EHD2,ARFGAP1,PARD3,RAB11FIP1
0.042654028	5.79E-06	Regulation of actin cytoskeleton	211	ITGB4,ARHGAP35,RAF1,SSH3,PXN,ARHGEF12,MAP2K2,DOCK1,CRK
0.043956044	5.95E-05	Chemokine signaling pathway	182	PLCB4,RAF1,PXN,GSK3A,LYN,AKT3,CRK,PARD3
0.055276382	0.00039212	Focal adhesion	199	ITGB4,ARHGAP35,RAF1,PXN,AKT3,PARVA,FLNB,DOCK1,CRK,TLN1,BAD
0.048	0.006568494	Spliceosome	125	HNRNPA1,TRA2A,DDX42,SF3B1,SRSF9,WBP11
0.053435115	0.011300642	Tight junction	131	EXOC4,MPDZ,SYMPK,EPB41L1,AKT3,VAPA,PARD3
0.04040404	0.012974065	Toll-like receptor signaling pathway	99	AKT3,MAP2K2,MAP3K7,IRF3
0.046296296	0.016061785	T cell receptor signaling pathway	108	RAF1,NCK1,AKT3,MAP2K2,MAP3K7
0.05511811	0.018732136	Neurotrophin signaling pathway	127	RAF1,RPS6KA4,IRS1,AKT3,MAP2K2,CRK,BAD
0.035294118	0.020442434	Progesterone-mediated oocyte maturation	85	RAF1,AKT3,CDC27
0.044444444	0.046271498	Dilated cardiomyopathy	90	ITGB4,LMNA,EMD,TPM3

Supplementary Table 1. Summary of the KEGG pathway analysis of IAV-responsive phosphosites.
