# Supplementary material for: Phosphoproteomic-based kinase profiling early in influenza virus infection identifies GRK2 as antiviral drug target
Source: Nat Commun. 2018 Sep 11;9:3679. doi: 10.1038/s41467-018-06119-y (PMC6133941; doi:10.1038/s41467-018-06119-y)
Supplement: Supplementary file 3 — Description of Additional Supplementary Files [file 41467_2018_6119_MOESM3_ESM.pdf]

Legends for Supplementary datasets:

Supplementary Data 1. **Identification of chicken proteins present in samples subjected to phosphoproteomic analysis.** A quantitative proteomic analysis of chicken proteins present in virus stock and allantoic fluid from mock-infected eggs was performed. Identified proteins and their relative abundance are listed.

Supplementary Data 2. **Summary and raw data of phosphoproteomic analysis early in IAV infection.** Raw and analyzed data from the phosphoproteomics experiment are given in tabs 1-6: Tab 1. Median fold change of abundance for all phosphoproteins identified. Tab 2. All phosphoproteins identified with median log2 FC >= 0.5. Tab 3. All phosphoproteins identified with median log2 FC <= 0.5. Tab 4. Raw data for TiO2 enrichment methodology. Tab 5. Raw data for Ti+4-IMAC enrichment methodology. Tab 6. Raw data for total proteomic analysis.
